# A quantitative study of pathologists’ perceptions towards artificial intelligence-assisted diagnostic system

**DOI:** 10.1371/journal.pdig.0001052

**Published:** 2025-10-17

**Authors:** Zichen Ye, Qu Lu, Jiahui Wang, Yu Jiang, Peng Xue

**Affiliations:** 1 School of Health Policy and Management, Chinese Academy of Medical Sciences and Peking Union Medical College, Beijing, China; 2 School of Population Medicine and Public Health, Chinese Academy of Medical Sciences and Peking Union Medical College, Beijing, China; Jordan University of Science and Technology, JORDAN

## Abstract

The successful implementation of artificial intelligence-assisted diagnostic system (AIADS) in pathology relies not only on the maturity of AI technology but also on pathologists’ cognition and acceptance of AI. However, research on pathologists’ perceptions towards AIADS is limited. This study aims to explore pathologists’ knowledge, attitudes, and practice toward AIADS and identify key factors influencing their willingness to use it, providing insights for the effective integration of AI technology in pathology. An online, nationwide, cross-sectional survey is to investigate pathologists’ knowledge, attitudes and behavioral intention/practice regarding AIADS with a 5-point Likert scale. Descriptive analysis is used to present the results, while logistic regression examines factors influencing AIADS adoption. The mediating effect of attitude in the association between knowledge and behavioral intention is also explored. A total of 224 pathologists were surveyed, with 85 (37.9%) having used AIADS and 139 (62.1%) not using it. The mean scores for knowledge, attitude, and behavioral intention were 3.42 ± 0.97, 3.48 ± 0.44, and 3.47 ± 0.44, respectively. Pathologists who had used AIADS scored higher in knowledge, attitude, and behavioral intention, with clearer attitudes toward AIADS. Over 80% of pathologists supported the use of AIADS in clinical diagnostics, citing improved diagnostic speed and reduced workload as key reasons. The main concerns about AIADS were its diagnostic accuracy. Logistic regression analysis indicated that a greater likelihood of willingness to use AIADS was associated with not having used it before (OR=2.462, 95%CI 1.087-5.573), as well as with higher knowledge scores (OR=1.140, 95%CI 1.076-1.208) and more positive attitude scores (OR=1.119, 95%CI 1.053-1.189). Mediation analysis indicated an indirect path from knowledge to behavioral intention through attitude among individuals who have used AIADS, with the mediation effect accounting for 59.4%. In conclusion, most pathologists support the use of AIADS in clinical practice, but improvements in diagnostic performance are necessary. Enhancing pathologists’ knowledge, attitudes, and user experience is crucial for the broader adoption of AIADS.

## Author summary

This study explores pathologists’ perceptions of artificial intelligence-assisted diagnostic systems (AIADS), focusing on their knowledge, attitudes, and behavioral intention/practice towards AI technology in pathology. By conducting a nationwide survey, we aimed to identify factors influencing their willingness to adopt AIADS. The findings revealed that while a majority of pathologists supported AIADS, with many highlighting its potential to improve diagnostic speed and reduce workload, concerns about diagnostic accuracy persisted. Pathologists who had used AIADS generally displayed higher levels of knowledge, more positive attitudes, and a stronger willingness to use it. The study also showed that pathologists’ willingness to use AIADS was associated with their prior usage experience, knowledge, and attitudes toward AIADS. This research offers valuable insights into the factors that influence AI integration in pathology and emphasizes the need for improved education and user experience to enhance its adoption in clinical practice.

## Introduction

Artificial intelligence (AI) has advanced markedly in medical imaging [1,2], utilizing deep learning to rapidly analyze large-scale datasets and enhance diagnostic accuracy [3,4]. Its adoption is relatively mature in radiology, where data are highly standardized and diagnoses are predominantly image-based [5,6]. In contrast, pathology involves complex morphological and clinical integration, leading pathologists to adopt a more cautious stance toward AI’s role [7]. Although liability concerns are relevant to both specialities, pathologists are likely more sensitive to AI-related misdiagnosis risks because their diagnosis is the gold standard for many cancers and directly impacts treatment decisions and prognosis. Consequently, requirements for interpretability, workflow integration, and accuracy are substantially heightened in pathology. This disparity is reflected in the regulatory landscape: as of December 2024, radiology accounts for 76% of U.S. FDA-approved AI-based medical devices, while pathology represents fewer than 0.5% [8]. Currently, the potential applications of AI in pathology are becoming increasingly evident [9–11], particularly in improving diagnostic efficiency, accuracy, and reducing pathologists’ workload. An AI in digital pathology systematic review found a mean sensitivity of 96.3% and a mean specificity of 93.3% across various diseases [12]. However, the successful integration of AI depends not only on the maturity of the technology itself but also on pathologists’ cognition and acceptance of AI [13].

In recent years, an increasing number of studies have focused on investigating clinicians’ knowledge, attitude, and practice (KAP) regarding AI technologies [14,15], as well as the factors influencing their behavioral intention [16–18]. Research also indicates that the acceptance of AI varies across different medical fields [19,20]. For instance, clinicians in the radiology field generally exhibit higher acceptance of AI [21–23], while there are more concerns in the field of pathology [24]. However, studies on pathologists’ perceptions toward AI systems remain limited. Therefore, thoroughly investigating pathologists’ attitudes and needs regarding AI has become critical to driving the adoption of AI technology and optimizing diagnostic workflows in pathology.

Through a nationwide survey, this study aims to assess pathologists’ knowledge, attitudes, and behavioral intentions/practice regarding AI-Assisted Diagnostic System (AIADS) to identify key knowledge gaps and trust issues, thereby providing empirical evidence to address adoption challenges and guide future medical policy and AI development.

## Materials and methods

### Study design and survey

An online nationwide cross-sectional survey was conducted among pathologists in August 2024. Participants were recruited through a multi-pronged strategy: initial dissemination via a national multi-center cervical cancer cohort network (encompassing over 20 hospitals of varying tiers across China), supplemented by snowball sampling.

The questionnaire was divided into six sections, with about 10 items per section, and participants were unable to return to previous sections once they had proceeded. Each participant (identified by IP address) could only complete the survey once, and all required fields had to be filled out; incomplete responses could not be submitted. Additionally, a quality control question was used to ensure the participants’ responses were thoughtful and accurate during the final survey.

### Ethics statement

This study adhered to the Declaration of Helsinki and was approved by the Institutional Review Board of the Chinese Academy of Medical Sciences and Peking Union Medical College (CAMS and PUMC-IEC-2022–022). All participants provided written informed consent prior to their involvement in the study.

### Questionnaire development

The questionnaire on pathologists’ knowledge, attitudes, and behavioral intention/practice toward the AIADS was designed based on the research topic, with reference to previous literature [14,15,25,26]. Before the formal survey, a small-scale pilot study (n = 30) was conducted to assess the questionnaire’s clarity and appropriateness, and it was also reviewed by field experts to refine its validity and comprehensiveness.

The questionnaire consists of four sections: demographic information, knowledge, attitudes, and behavioral intentions/practice about AIADS. The detailed questionnaire is provided in S1 Appendix.

**1. Demographic information:** This section includes 11 items such as age, gender, workplace, ethnicity, education level, hospital level, professional title, and years of work experience.
**2. Knowledge about AIADS:** This section assesses pathologists’ knowledge of AIADS using 7 items with a 5-point Likert scale. The options range from “Very unfamiliar” to “Very familiar” scored from 1 to 5. This is followed by further questions on AIADS’s functions, diagnostic performance, advantages, and limitations.
**3. Attitude towards AIADS:** Before this section, a brief introduction to AIADS in pathology is provided, covering its functions, diagnostic performance, advantages, and limitations, to enhance pathologists’ understanding. Participants then respond to 11 items using a 5-point Likert scale (“Strongly disagree” to “Strongly agree”), with scores ranging from 1 to 5. Subsequently, pathologists are asked about their trust in AIADS, concerns regarding diagnostic errors, and issues related to responsibility.
**4. Behavioral intention/practice regarding AIADS:** This section includes a Likert scale with 5 items assessing pathologists’ intention to use AIADS, scored from 1 to 5. Participants are also asked whether they have ever used AIADS in the field of pathology before participating in this survey, followed by further questions based on their responses (Yes/No). Finally, the role of AIADS in pathology and potential barriers to its wider adoption are explored.

The reliability of the scales was assessed using Cronbach’s α. The results showed that the Cronbach’s α coefficients for the three scales were 0.949, 0.720, and 0.950, respectively, all exceeding the acceptable threshold of 0.7 [27], indicating good internal consistency.

### Sample size calculation

As a convenience sampling method was employed in this study, a formal sample size calculation was not performed a priori during the initial design phase. The objective was to maximize the number of collected responses. To assess the precision of the estimates achieved with the obtained sample, a post-hoc analysis was conducted to compute the width of the confidence interval (margin of error).

Using the “Confidence Intervals for One Proportion” module in PASS 15.0 software, the analysis was performed with the following parameters: the primary outcome was behavioral intention, the proportion was 0.661, the achieved sample size was 224, and a two-tailed alpha level of 0.05 was applied. The result yielded a 95% confidence interval of 0.661 (0.595, 0.722). The width of this confidence interval was 0.128, corresponding to a margin of error of ±0.064.

### Statistical analysis

This study used SPSS 29.0 for statistical analysis, with Origin 2024 and Adobe Illustrator 2023 for data visualization. A significance level of *α* = 0.05 (two-tailed) was applied. For continuous variables, normally distributed data were expressed as mean±standard deviation (Mean±SD) and analyzed using t-tests or ANOVA. Categorical data were presented as frequencies (percentages) and analyzed using Chi-square tests (χ^2^).

The knowledge, attitude, and behavioral intention scales were treated as continuous variables, with the average scores and standard deviations for each scale and item calculated (higher scores indicating more positive outcomes, with assigning reverse scores to negative items). Logistic regression was employed to analyze behavioral intention, with results evaluated using odds ratios (OR) and 95% confidence intervals (CI). Behavioral intention was categorized into willingness and unwillingness to use AIADS based on a cutoff score of 20 out of 25. This threshold was selected a priori as it represents a consistent “Agree” response (4/5 points per item), ensuring a definitive positive intention, and yielded a balanced distribution for analysis. Variables with a univariate P-value of <0.20 were included in the multivariate analysis to explore influencing factors. Mediation analysis was performed using Hayes’ SPSS macro PROCESS Version 4.0 (models 4 and 58) [28] to examine the associations among knowledge (independent variable), attitude (mediator), and behavioral intention (dependent variable), controlling for demographic characteristics. Bootstrapping was applied with 5000 resamples to ensure the stability of the mediation results. Subgroup analyses were conducted based on whether participants had used the system before.

## Results

### Demographic information

This study collected a total of 235 questionnaires, of which 224 were valid, yielding a validity rate of 95.3%. The participants were pathologists from over 120 hospitals across 27 provinces, municipalities, and autonomous regions in China, covering 7 geographic regions. The detailed distribution is shown in S1 Fig. Among the participants, 73.2% (n = 164) were female, and the average age was 40.34 ± 8.18 years. Of the participants, 37.9% (n = 85) had used AIADS, while 62.1% (n = 139) had not. Detailed results can be found in Table 1.

**Table 1:** Demographic characteristics of participants (N = 224).

Characteristics		N (%)	Knowledge	Attitude	Behavioral intention
			Mean ± SD		
Gender					
	Male	60 (26.8)	3.60 ± 0.96	3.32 ± 0.60	3.92 ± 0.78
	Female	164 (73.2)	3.35 ± 0.97	3.29 ± 0.54	3.98 ± 0.66
	T		1.738	0.389	0.635
	P		0.084	0.698	0.526
Ethnicity					
	Han	208 (92.9)	3.43 ± 0.98	3.32 ± 0.57	3.98 ± 0.69
	Others	16 (7.1)	3.33 ± 0.87	3.14 ± 0.43	3.73 ± 0.64
	T		0.378	1.288	1.448
	P		0.289	0.094	0.858
Age					
	Mean ± SD	40.34 ± 8.18			
	<30	19 (8.5)	3.47 ± 1.13	3.26 ± 0.60	3.83 ± 0.88
	30-39	90 (40.2)	3.47 ± 0.99	3.34 ± 5.25	3.95 ± 0.71
	40-49	84 (37.5)	3.33 ± 0.99	3.27 ± 0.62	3.99 ± 0.64
	≥50	31 (13.8)	3.48 ± 0.79	3.29 ± 0.45	4.05 ± 0.66
	F		0.361	0.263	0.419
	P		0.781	0.852	0.739
Education level					
	College degree and below	12 (5.4)	3.68 ± 0.86	3.49 ± 0.23	4.12 ± 0.53
	Bachelor degree	157 (70.1)	3.37 ± 0.97	3.30 ± 0.58	3.97 ± 0.68
	Master degree and above	55 (24.5)	3.51 ± 0.00	3.31 ± 0.55	3.92 ± 0.74
	F		0.861	0.690	0.408
	P		0.424	0.503	0.666
Hospital level					
	Primary hospital and below	10 (4.5)	3.90 ± 1.26	3.44 ± 0.57	4.20 ± 0.43
	Secondary hospital	80 (35.7)	3.27 ± 0.90	3.31 ± 0.51	3.92 ± 0.65
	Tertiary hospital	134 (59.8)	3.47 ± 0.98	3.30 ± 0.59	3.97 ± 0.73
	F		2.337	0.271	0.739
	P		0.099	0.763	0.479
Title					
	Resident physician	71 (31.7)	3.48 ± 1.05	3.28 ± 0.62	3.92 ± 0.78
	Attending physician	106 (47.3)	3.34 ± 0.94	3.31 ± 0.49	3.95 ± 0.60
	Associate chief physician and above	47 (21.0)	3.50 ± 0.93	3.35 ± 0.60	4.07 ± 0.73
	F		0.653	0.209	0.735
	P		0.522	0.812	0.480
Years doing pathology					
	Mean ± SD	11.86 ± 7.77			
	≤5	48 (21.4)	3.29 ± 1.14	3.22 ± 0.59	3.86 ± 0.87
	6-10	54 (24.1)	3.48 ± 0.85	3.28 ± 0.53	3.99 ± 0.62
	11-15	57 (25.5)	3.42 ± 0.97	3.32 ± 0.54	3.88 ± 0.54
	>15	65 (29.0)	3.46 ± 0.94	3.39 ± 0.57	4.09 ± 0.70
	F		0.394	0.967	1.379
	P		0.758	0.409	0.250
Specialized fields^#^					
	Cytopathology	55 (24.6)	3.77 ± 0.89	3.36 ± 0.57	3.97 ± 0.71
	Histopathology	32 (14.3)	3.41 ± 0.99	3.26 ± 0.59	3.91 ± 0.68
	Pathology	137 (61.1)	3.28 ± 0.97	3.30 ± 0.55	3.98 ± 0.69
	F		5.196	0.343	0.136
	P		0.006	0.710	0.873
Have you ever used AIADS*					
	Yes	85 (37.9)	3.91 ± 0.86	3.55 ± 0.50	4.02 ± 0.84
	No	139 (62.1)	3.12 ± 0.92	3.15 ± 0.53	3.93 ± 0.58
	T		6.411	5.561	0.989
	P		<0.001	<0.001	0.183

^#^ Please select “Pathology” if your specialized field does not differentiate between histopathology and cytopathology. Otherwise, please select the specific subspecialty.

* The original sentence is “Have you ever used AIADS in the field of pathology before participating in this survey?”.

### Result of Likert scale about AIADS

**Fig 1:**
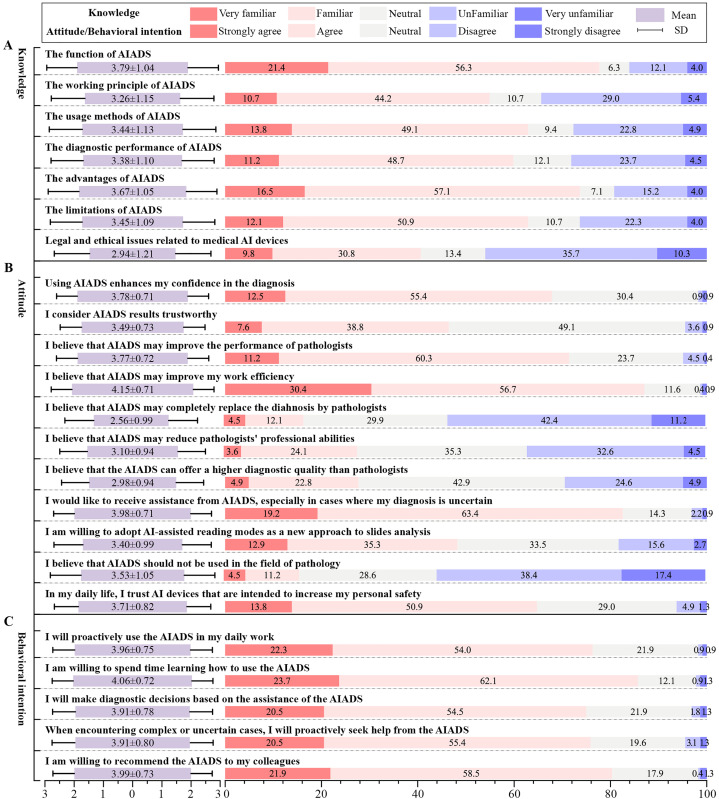
The result of Likert scale about AIADS. A. Knowledge about AIADS; B. Attitude towards AIADS; C. Behavioral intention about AIADS. AIADS, artificial intelligence-assisted diagnostic system.

This study assessed participants’ knowledge (7 items), attitudes (11 items), and behavioral intentions (5 items) towards AIADS using a Likert scale. Detailed scores are presented in Fig 1 and S1 Table. The Mean±SD for the knowledge, attitude, and behavioral intention were 3.42 ± 0.97, 3.48 ± 0.44, and 3.47 ± 0.44, respectively. For the knowledge scale, only 40.6% (n = 91) of participants knew the legal and ethical issues surrounding medical AI devices. For the remaining items, more than 50% of participants indicated some level of awareness of the relevant topics, with detailed data presented in Fig 1A.

Regarding attitudes, over 80% of participants agreed with the following statements: “AIADS can enhance my work efficiency” and “ I would like to receive assistance from AIADS, especially in cases where my diagnosis is uncertain. “However, only 16.5% (n=37) of participants agreed with the statement, “AIADS will completely replace the diagnosis by pathologists.” Additionally, 55.8% (n = 125) of participants disagreed with the statement, “AIADS should not be used in the field of pathology.” Detailed data are shown in Fig 1B.

About behavioral intentions, over 70% of pathologists expressed an intention to use AIADS for all 5 items, with fewer than 5% indicating they were unwilling to use AIADS. Around 17%-22% of pathologists remained neutral on these items. Detailed results are shown in Fig 1C.

### Difference analysis results of the Likert scale for AIADS

**Fig 2:**
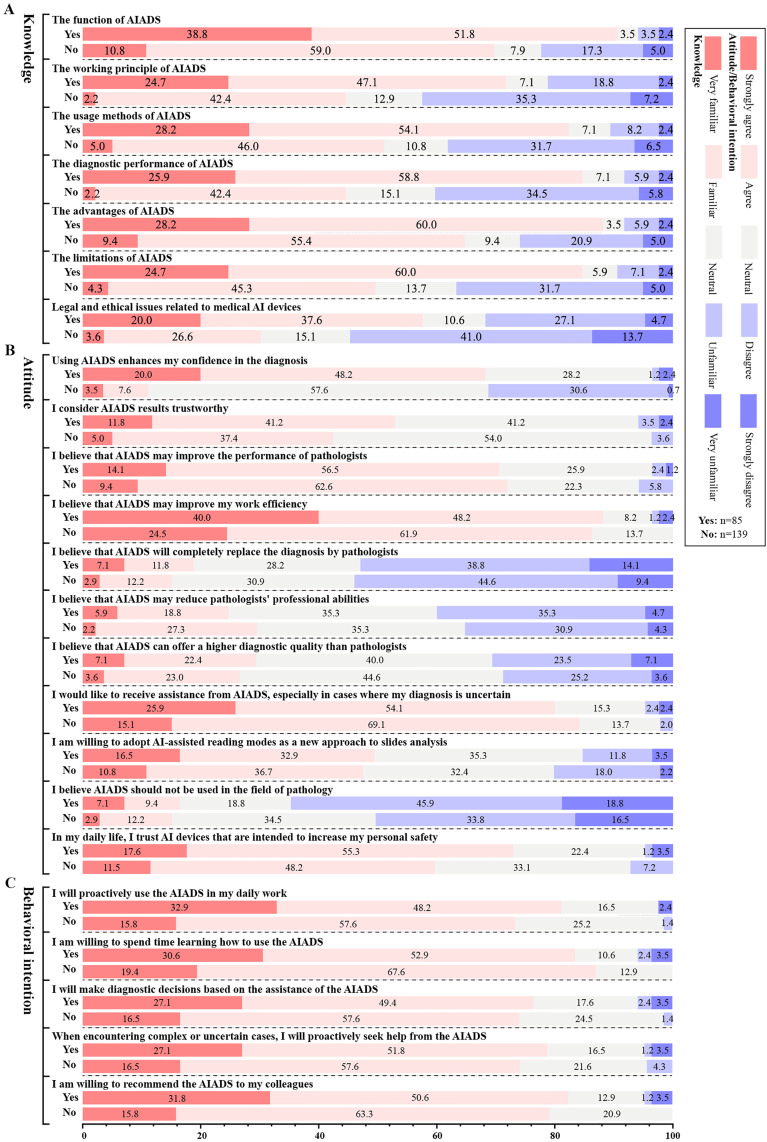
Subgroup analysis results of the Likert scale for AIADS. A. Knowledge about AIADS; B. Attitude towards AIADS; C. Behavioral intention about AIADS. AIADS, artificial intelligence-assisted diagnostic system; Yes: I have ever used AIADS in the field of pathology before participating in this survey; No: I have not ever used AIADS in the field of pathology before participating in this survey.

The results of the demographic difference analysis for knowledge, attitude, and behavioral intention are presented in Table 1. Knowledge and attitude showed significant differences based on prior use of AIADS (*P* < .05). Specifically, those who had used AIADS generally scored higher. Subgroup analyses based on AIADS usage (Yes/No) for knowledge, attitude, and behavioral intention are shown in Fig 2 and S2 Table. Except for one item, the scores for all other items were higher in the group that had used AIADS. However, statistically significant differences were observed for all items in the Knowledge scale (*P* < .001), while the overall mean score of the attitude scale also showed a statistically significant difference.

In the knowledge scale, the group that has used AIADS demonstrates a better understanding of AIADS, with higher proportions of “very familiar” and “familiar” responses across all items compared to the group that has not used AIADS (see Fig 2A). In the attitude scale, the group that has used AIADS shows a higher proportion of “strongly agree” and “agree” responses only for the item “Using AIADS enhances my confidence in the diagnosis”, while no significant differences are observed in other items (see Fig 2B). In the behavioral intention scale, the group that has used AIADS exhibits higher proportions of “strongly agree” responses across all items compared to the non-user group, although the overall differences between the two groups are not large (see Fig 2C).

### Detailed statements of knowledge, attitude and behavioral intention/practice

**Fig 3:**
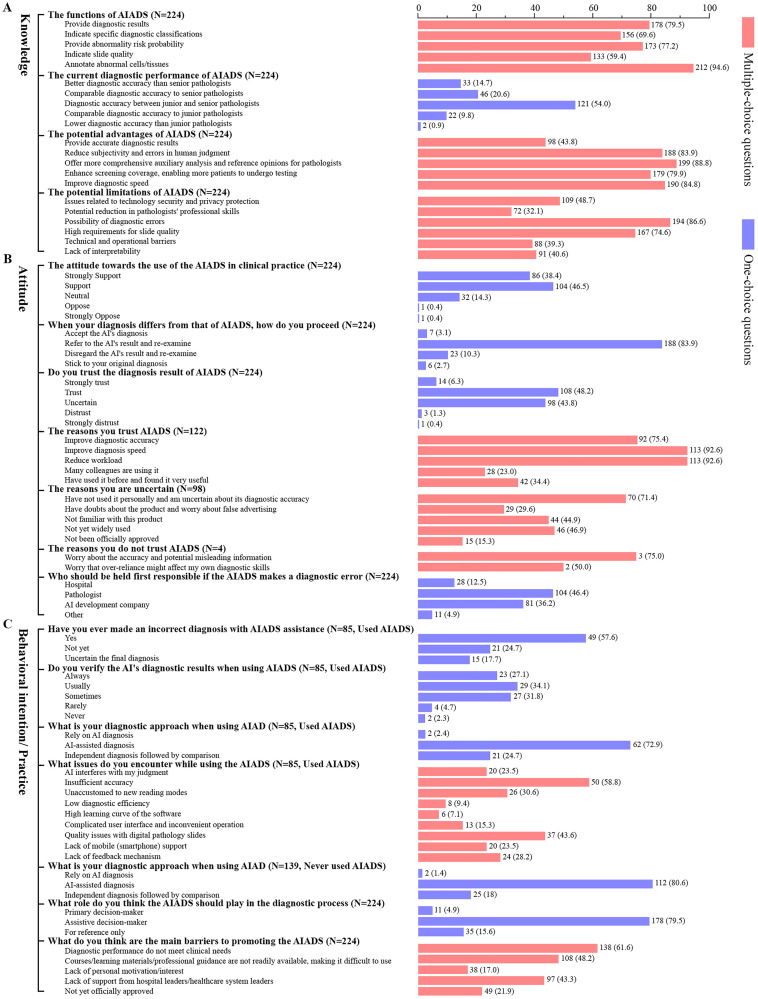
Detailed statements of knowledge, attitude and behavioral intention/practice related to AIADS. A. Detailed statements of knowledge about AIADS; B. Detailed statements of attitude towards AIADS; C. Detailed statements of behavioral intention/practice about AIADS. AIADS, artificial intelligence-assisted diagnostic system.

Fig 3 provides detailed responses to questions regarding knowledge, attitude, and behavioral intention/practice related to AIADS. Regarding knowledge, only 43.8% (n = 93) of participants recognized that AIADS could “provide accurate screening results” as one of its advantages. Regarding limitations, participants primarily identified two concerns: “potential diagnostic errors” (n=194, 86.6%) and “high requirements for slide quality” (n = 167, 74.6%). Detailed results are presented in Fig 3A.

In terms of attitude, over 80% of participants supported the use of AIADS in clinical diagnosis. When AI conclusions conflicted with their own diagnosis, 83.9% of participants chose to “reference the AI’s results and re-examine” the case. Regarding trust, 54.5% (n = 122) of participants trusted AI’s results, primarily because it improves diagnostic speed (n = 113, 92.6%) and reduces workload (n = 113, 92.6%). However, 43.8% (n = 98) were uncertain about trusting AI, mainly due to “lack of personal experience and uncertainty about diagnostic accuracy” (n = 70, 71.4%). Only 1.7% (n = 4) expressed distrust in AI. Additionally, 82.1% (n = 184) were concerned that AIADS could lead to diagnostic errors. Regarding responsibility for diagnostic errors, the primary attributions were to the pathologist (n = 104, 46.4%), AI developers (n = 81, 36.2%), and the hospital (n = 28, 12.5%), with specific data shown in Fig 3B.

Regarding behavioral intention/practice, we surveyed participants who had used (n = 85) and not used (n = 139) AIADS on various questions, with detailed data shown in Fig 3C. Among those who had used AIADS, 57.6% (n = 49) reported encountering issues with diagnostic inaccuracies. Additionally, over 90% (n = 79) of participants verified AI results during the diagnostic process. When using AIADS, 72.9% (n = 62) considered it an auxiliary tool. The main issues reported by users included “insufficient accuracy”, “digital pathology slide quality”, and “unaccustomed to new reading mode”. Regarding the evaluation of AIADS, 97.6% (n = 83) of participants found the system helpful in their work, particularly in marking abnormal cells/tissues and improving diagnostic speed, as detailed in S3 Table. On the role of AIADS, 79.5% (n = 178) of participants believed that AIADS should serve as a decision-support tool rather than replacing pathologist decision-making.

### Logistic regression results of influencing factors of behavioral intention

A total of 148 participants expressed a willingness to use AIADS. Multivariate logistic regression identified five factors with significant associations with the intention to use AIADS out of the seven factors analyzed, as detailed in Table 2. Specifically, Han ethnicity (OR=4.595, 95%CI 1.236-17.089), no prior usage experience (OR=2.462, 95%CI 1.087-5.573), higher knowledge scores (OR=1.140, 95%CI 1.076-1.208), and more positive attitude scores (OR=1.119, 95%CI 1.053-1.189) were associated with greater willingness. In contrast, working in a tertiary hospital was associated with reduced willingness (OR=0.076, 95%CI 0.006-0.943). Furthermore, subgroup analysis stratified by prior AIADS usage indicated that the positive associations of knowledge (OR=1.496, 95%CI 1.016-2.020) and attitude (OR=2.690, 95%CI 1.351-5.353) with intention to use were more pronounced among participants with prior AIADS experience.

**Table 2:** Logistic regression results of influencing factors of behavioral intention.

Variable		B	S.E.	Wald	P	OR (95% CI)
Ethnicity (Others for reference)						
	Han	1.525	0.67	5.179	0.023	4.595 (1.236-17.089)
Hospital level (Primary hospital and below for reference)						
	Secondary hospital	-2.415	1.297	3.466	0.063	0.089 (0.007-1.136)
	Tertiary hospital	-2.572	1.282	4.024	0.045	0.076 (0.006-0.943)
Title (Resident physician for reference)						
	Attending physician	0.247	0.428	0.333	0.564	1.280 (0.553-2.964)
	Associate chief physician and above	0.469	0.585	0.643	0.422	1.598 (0.508-5.027)
Years doing pathology (≤5 for reference)						
	6-10	-0.833	0.501	2.763	0.096	0.435 (0.163-1.161)
	11-15	-0.496	0.543	0.835	0.361	0.609 (0.210-1.765)
	>15	0.341	0.576	0.350	0.554	1.406 (0.455-4.352)
Have you ever used AIADS* (Yes for reference)						
	No	0.901	0.417	4.670	0.031	2.462 (1.087-5.573)
Knowledge (Sum score)		0.131	0.029	19.874	<0.001	1.140 (1.076-1.208)
Attitude (Sum score)		0.112	0.031	13.098	<0.001	1.119 (1.053-1.189)

* The original sentence is “Have you ever used AIADS in the field of pathology before participating in this survey?”.

### The result of mediation model

The results of the mediation analysis are presented in S4 and S5 Tables. In both the unadjusted model (Model 1) and the model adjusted for demographic information (Model 2), the indirect pathways were statistically significant (all *P* < .05), with the proportion of the total association accounted for by the indirect pathway being 17.0% and 18.4%, respectively. After including the variable “Have you ever used AIADS” (Model 3), the indirect pathway was no longer significant (0.0315, 95%CI -0.0107-0.0783), and the proportion of the total association accounted for by the indirect pathway decreased to 13.0%. Further subgroup analysis based on “Have you ever used AIADS” showed that, after adjusting for demographic information (Model 2), the indirect pathway through attitude was not statistically significant for participants who had not used AIADS (*P* > 0.05). In contrast, for participants who had used AIADS, a significant indirect pathway was observed, accounting for 59.4% of the total association, as detailed in S6 and S7 Tables.

## Discussion

### Principal results

This study surveyed 224 pathologists regarding their knowledge, attitudes, and behavioral intentions toward AIADS. The results show that most pathologists have a certain level of knowledge about AIADS, hold positive attitudes, and express a willingness to use it. Participants who have used AIADS scored higher in knowledge, attitude, and behavioral intention than those who have not, and their attitudes toward AIADS were clearer. The primary reasons for supporting the use of AIADS in clinical diagnosis were its potential to improve diagnostic efficiency. However, the main reason why participants distrust AIADS and its limitations focuses on “insufficient diagnostic accuracy”. Logistic regression analysis indicated that behavioral intention was associated with key factors, including ethnicity, hospital level, AIADS usage experience, knowledge, and attitude. Mediation analysis further suggested that, among users of AIADS, the association between knowledge and behavioral intention was partially explained by attitude.

Pathologists generally support the application of AIADS in pathology, believing they can enhance diagnostic efficiency and provide valuable supplementary information. Our results align with Sarwar et al. [13], showing generally positive respondent attitudes toward AI in diagnostic pathology. And studies have shown that AI demonstrates excellent diagnostic performance in pathology [12], improving diagnostic accuracy and reducing inter-observer variability [29]. The cervical cytology AI system developed by Xue et al. significantly improved the sensitivity and specificity of junior cytopathologists (0.857 vs 0.657, 0.840 vs 0.737; both *P* < .001), while also reducing reading time (218 seconds vs 30 seconds; *P* < .001) [30]. However, this study found that despite pathologists’ support for AI, there remain concerns about its diagnostic accuracy. Currently, the effectiveness of AI in medical applications remains controversial [31–33], particularly in the field of pathology, where, despite rapid development, several challenges persist [34]. The quality of pathology slides is influenced by factors such as sample preparation, staining, and other variables [35,36], while AI systems rely heavily on large annotated datasets, which are often lacking [37]. Additionally, annotation is difficult, and there is considerable variability between experts [33], which leads to poorer performance of AI in rare cases. AI training often uses experienced clinicians as the “gold standard”, but even their accuracy is limited [38], especially in complex cases, where AI may perform worse than seasoned clinicians. These issues constrain the development of AI in pathology, and improvements in diagnostic accuracy are still needed.

Research showed that pathologists generally have a high willingness to use AIADS, although some remain cautious, indicating that various factors influence their behavioral intention. The study found that pathologists in tertiary hospitals have a lower acceptance of AIADS compared to those in primary hospital, likely due to differences in resources and value perceptions. This study found no significant impact of gender, work experience, or age on the willingness to use AIADS. A review by Lambert et al. [19] indicated that gender does not significantly affect acceptance, while the impact of age and experience varies across studies. This study found that higher knowledge levels positively influence willingness to use AI, consistent with the Technology Acceptance Model, which suggests that attitudes and intentions toward new technology are largely shaped by understanding [39]. This finding is consistent with existing research indicating that attitude is a significant factor influencing the willingness to use AI [16–18]. In practice, clinicians’ willingness to adopt AI is shaped by various interrelated factors. Understanding these factors is key to developing effective strategies and training programs that boost AI adoption.

This study found that pathologists who had not used AIADS expressed a stronger willingness to use such systems compared to those with prior experience—a result contrary to conventional expectations. As shown in multivariable analysis (see S8 Table), after adjusting for knowledge and/or attitude scores, the association between prior usage and willingness to use reversed significantly, indicating that non-users had a notably higher willingness. This suggests that hands-on experience may indirectly influence behavioral intention through its effects on cognition and attitude. Specifically, although the user group scored higher in knowledge and attitude, there was no significant difference in the behavioral intention scores between the two groups (see Table 1). Notably, the non-users exhibited a greater difference in the scores of “behavioral intention-knowledge” (0.81 vs. 0.11) and “behavioral intention-attitude” (0.78 vs. 0.47), indicating that, despite having a weaker foundation in cognition and attitude, their willingness to use was relatively stronger.

Possible reasons for this phenomenon include performance and reliability issues with AIADS in real-world practice. This study found that over half of the users encountered diagnostic errors, and most felt the need to frequently verify results, indicating concerns regarding accuracy and slide quality. Additionally, insufficient integration with clinical workflows may increase operational burdens, as research shows that users are unaccustomed to the new reading methods. Furthermore, the lack of training and technical support further hinders the user experience [40,41]. The higher willingness among non-users may stem from anticipation and “technology optimism bias” [42,43], though its translation into sustained usage depends heavily on the system’s actual performance and integration. Therefore, promoting AIADS requires not only advocacy but also focused efforts on enhancing algorithmic accuracy, improving workflow integration, and establishing systematic training and feedback mechanisms to convert initial willingness into sustained adoption. Future implementation strategies should prioritize real-world reliability, usability, and user experience.

This study examined the mediating role of attitude in the association between knowledge and behavioral intention. The results indicate that attitude served as a significant mediator in this association in the initial models. However, when prior experience with AIADS was included as a covariate, the overall mediation effect was no longer significant, suggesting that prior use may alter the pathway linking knowledge, attitude, and intention. Subgroup analysis further indicated that this moderating pattern reflects population heterogeneity: among participants without prior AIADS experience (accounting for 62.1% of the sample), attitude was not a significant mediator. In contrast, among those with AIADS experience, attitude showed a strong and significant mediating role. These findings suggest that hands-on experience with AIADS may influence the mediating role of attitude, potentially facilitating the association between knowledge and intention via stronger attitude. This highlights that fostering AI adoption may depend not only on increasing knowledge, but also on building practical experience and attitude—key factors that may help translate knowledge into clinical use.

Studies indicate that pathologists lack sufficient legal and ethical knowledge regarding AI. Research consistently shows that clinicians are underprepared in this area [44–46]. The main reasons include: rapid development of AI technology outpacing medical education, which lacks relevant ethics and legal courses; limited understanding of AI’s workings and algorithms, making it difficult for clinicians to identify ethical risks. To address these issues, we recommend implementing systematic and institutional educational interventions at multiple levels. First, AI ethics and legal courses should be integrated into core medical education, particularly within standardized residency training and continuing education programs for pathologists, emphasizing case-based learning and practical application. Second, professional associations, such as the Chinese Society of Pathology, should spearhead the development of official guidelines and industry standards to provide clear behavioral protocols for practitioners and address knowledge gaps and legal uncertainties at a macro level. Finally, health authorities should introduce regulations that clarify accountability frameworks for AI applications in healthcare and promote the certification and implementation of continuing education courses focusing on AI functionality, data privacy, and practical methods for prudent use of AI-assisted decision-making.

The application of AIADS in clinical practice may lead to diagnostic errors, making responsibility attribution a complex issue [47]. This study indicates that physicians assign responsibility for errors in a descending order to pathologists, AI developers, and hospitals, aligning with current findings [26,48] and suggesting that a singular accountability model is inadequate. Accordingly, a shared responsibility model is proposed to achieve fair and effective risk allocation. Within this framework, clinicians, as final decision-makers, are responsible for critically verifying AI outputs and exercising independent judgment; AI developers must ensure algorithmic transparency, explainability, and provide comprehensive validation reports; and healthcare institutions should undertake technical validation and internal oversight to ensure appropriate use of AI systems within controlled environments. This model aligns each party’s responsibilities with their role in the AI application chain, offering a feasible approach for future legal traceability and risk management. To operationalize this model, legislative measures should be advanced to clarify legal responsibility distribution in AI-assisted healthcare—for instance, by leveraging medical device regulations to define the legal status of AI as a “medical device”. Simultaneously, liability attribution and compensation mechanisms must be refined to reflect the multi-stakeholder nature of AI-related incidents. Furthermore, improving the interpretability and decision-making transparency of AI systems will not only strengthen physicians’ trust but also facilitate rapid attribution and root-cause analysis when errors occur.

This study employed a nationwide survey covering hospitals of different tiers across various regions in mainland China, the demographic characteristics of which closely mirrored those of large-scale national surveys [49,50], enhancing the representativeness of our sample. By investigating pathologists’ attitudes toward AIADS, it offers valuable insights for promoting the application of AI in healthcare and deepens the understanding of pathologists’ interactions with AIADS and their needs. However, several limitations should be noted. First, due to the convenience sampling and online recruitment strategy, a precise response rate could not be calculated, which may introduce selection bias. Second, the treatment of ordinal Likert scale responses as continuous variables, which assumes equal intervals between categories, an assumption that may not fully reflect participants’ interpretations of the scale. Third, the cross-sectional design precludes causal inference, and reverse causality remains a possible explanation for the observed associations. Additionally, the sample is primarily drawn from secondary and tertiary hospitals, with fewer pathologists from primary and below hospitals, which may introduce selection bias and fail to fully reflect the conditions in grassroots healthcare settings. Finally, the small sample size for subgroup analyses may lead to unstable results. Future studies could employ a probabilistic sampling approach, expand the sample size—particularly among pathologists in primary care settings—and incorporate long-term follow-up to further validate the findings and support the development of AI in the healthcare sector.

## Conclusions

The vast majority of pathologists hold a positive attitude toward using AIADS and are willing to adopt them. However, the diagnostic performance of AIADS needs further improvement. Pathologists’ knowledge, attitudes, and user experience are significantly associated with their acceptance of AIADS. It is recommended that future efforts focus on providing AI training for clinicians to enhance their trust and understanding of AI technology, thereby promoting its integration into clinical practice.

## Supporting information

S1 Appendix Questionnaire. (DOCX)

S1 Fig Geographic distribution of survey participants (N = 224). (DOCX)

S1 Table The score of Likert scale about AIADS (N = 224). (DOCX)

S2 Table Subgroup analysis results of the Likert scale score for AIADS (N = 224). (DOCX)

S3 Table The responses of participants who have used AIADS to behavioral intention/practice. (DOCX)

S4 Table Testing for mediation model. (DOCX)

S5 Table The result of direct effect and mediation effect. (DOCX)

S6 Table Testing for mediation model with subgroups. (DOCX)

S7 Table The result of direct effect and mediation effect with subgroups. (DOCX)

S8 Table The association between AIADS usage and behavioral intention. (DOCX)

## Abbreviations

AI: Artificial intelligence
AIADS: AI-Assisted Diagnostic System
CI: confidence interval
DL: deep learning
KAP: knowledge, attitude, and practice
OR: odds ratio
SD: standard deviation
