# Supplementary material for: A quantitative study of pathologists’ perceptions towards artificial intelligence-assisted diagnostic system
Source: PLOS Digit Health. 2025 Oct 17;4(10):e0001052. doi: 10.1371/journal.pdig.0001052 (PMC12533903; doi:10.1371/journal.pdig.0001052)
Supplement: S1 Appendix — (DOCX) [file pdig.0001052.s001.docx]

# Questionnaire

**Investigation of Pathologists' Knowledge, Attitudes, and Practices Regarding the application of Artificial Intelligence****-Assisted Diagnostic Systems in Pathology**

Dear participants,

Hello, we are a research team from the Chinese Academy of Medical Sciences and Peking Union Medical College. Before you decide whether to take part in this study, please read the following carefully:

We are conducting this survey to understand pathologists' knowledge, attitude, and behavioral intention/practice regarding Artificial Intelligence-assisted Diagnostic System (AIADS), with the aim of providing a theoretical basis for the promotion of such systems.

This survey will take approximately 5-10 minutes of your time. The questionnaire will be completed anonymously and the information you provide will be kept strictly confidential and the results will be used for scientific research only. This study is voluntary and you have the right to choose to participate or not to participate. We sincerely appreciate your cooperation and support!

**Informed consent.**

○ I agree to participate in this survey

○ I do not agree to participate in this survey (end of the questionnaire)

**A. Basic Demographic Information**

**A1.** Your gender: ○female ○male

**A2.** Your age: ____________

**A3.** Your ethnicity: ____________

**A4.** Your place of work: ____________ (Province - City)

**A5.** The level of your hospital: ○Primary hospital and below ○Secondary hospital ○Tertiary hospital

**A6.** Your education level: ○College degree or below ○Bachelor degree ○Master degree ○Doctoral degree

**A7.** Your professional title: ○Resident physician ○Attending physician ○Associate chief physician ○Chief physician

**A8.** How long have you been engaged in pathology? (since resident)? ___ year(s) ____ month(s)

**A9.** What is your specialized field of pathology? ○Cytopathology ○Histopathology ○Pathology

**A10.** Have you ever used AIADS in the field of pathology before participating in this survey?

○Yes ○No

**B Knowledge about AIADS**

**B1.** *To evaluate your understanding of* AIADS*, please choose the one that is most in line you’re your actual situation:* *Very unfamiliar, Unfamiliar, Neutral, Familiar, or Very familiar.*

| Items | Very unfamiliar | Unfamiliar | Neutral | Familiar | Very familiar |
| --- | --- | --- | --- | --- | --- |
| ① The function of AIADS. |  |  |  |  |  |
| ② The working principle of AIADS. |  |  |  |  |  |
| ③ The usage methods of AIADS. |  |  |  |  |  |
| ④ The diagnostic performance of AIADS. |  |  |  |  |  |
| ⑤ The advantages of AIADS. |  |  |  |  |  |
| ⑥ The limitations of AIADS. |  |  |  |  |  |
| ⑦ Legal and ethical issues related to medical AI devices |  |  |  |  |  |

**B2.** What do you think are the functions of AIADS? **(Select all that apply)**

① Provide diagnostic results (normal/abnormal)

② Indicate specific diagnostic classifications

③ Provide abnormality risk probability

④ Indicate slide quality

⑤ Annotate abnormal cells/tissues

⑥ Other: ______

**B3.** How do you think the current diagnostic performance of AIADS?

① Better diagnostic accuracy than senior pathologists (with 5 or more years of experience).

② Comparable diagnostic accuracy to senior pathologists.

③ Diagnostic accuracy between junior pathologists (with less than 1 year of experience) and senior pathologists.

④ Comparable diagnostic accuracy to junior pathologists.

⑤ Lower diagnostic accuracy than junior pathologists.

**B4.** What do you think are the potential advantages of AIADS? **(Select all that apply)**

① Provide accurate diagnostic results

② Reduce subjectivity and errors in human judgment

③ Offer more comprehensive auxiliary analysis and reference opinions for pathologists

④ Enhance screening coverage, enabling more patients to undergo testing

⑤ Improve diagnostic speed

⑥ Other: ___________________

**B5.** What do you think are the potential limitations of AIADS? **(Select all that apply)**

① Issues related to technology security and privacy protection

② Potential reduction in pathologists' professional skills

③ Possibility of diagnostic errors

④ High requirements for slide quality

⑤ Technical and operational barriers

⑥ Lack of interpretability

⑦ Other: ___________________

**C.** **Attitude about AIADS**

*Artificial Intelligence-Assisted Diagnostic Systems (AIADS) in Pathology: This system utilizes artificial intelligence technologies to assist pathologists in diagnosing diseases based on cellular or tissue pathology findings. Current research on AIADS demonstrates that their diagnostic performance is highly promising, surpassing that of less experienced pathologists and comparable to that of more experienced ones. The use of AI in diagnosis significantly enhances both the accuracy and efficiency of medical decision-making. However, there are still several risks associated with AI products, including concerns about transparency, fairness, and ethical and legal issues.*

**C1.** Please rate your attitude of the AIADS by selecting the option that best matches your actual situation: strongly disagree, disagree, neutral, agree, or strongly agree.

| Items | Strongly disagree | Disagree | Neutral | Agree | Strongly agree |
| --- | --- | --- | --- | --- | --- |
| ① Using AIADS enhances my confidence in the diagnosis. |  |  |  |  |  |
| ② I consider AIADS results trustworthy. |  |  |  |  |  |
| ③ I believe that AIADS may improve the performance of pathologists |  |  |  |  |  |
| ④ I believe that AIADS may improve my work efficiency. |  |  |  |  |  |
| ⑤ I believe that AIADS will completely replace the diagnosis by pathologists. |  |  |  |  |  |
| ⑥ I believe that AIADS may reduce pathologists' professional abilities. |  |  |  |  |  |
| ⑦ I believe that AIADS can offer a higher diagnostic quality than pathologists. |  |  |  |  |  |
| ⑧ I would like to receive assistance from AIADS, especially in cases where my diagnosis is uncertain. |  |  |  |  |  |
| ⑨ I am willing to adopt AI-assisted reading modes as a new approach to slides analysis. |  |  |  |  |  |
| ⑩ I believe AIADS should not be used in the field of pathology. |  |  |  |  |  |
| In my daily life, I trust AI devices that are intended to increase my personal safety (e.g. smoke detector, lane keeping assist system). |  |  |  |  |  |

**C2.** What is your attitude towards the use of the AIADS in clinical practice?

① Strongly Support ② Support ③ Neutral ④ Oppose ⑤ Strongly Oppose

**C3.** When your diagnosis differs from that of AIADS, how do you proceed?

① Accept the AI's diagnosis

② Refer to the AI's result and re-examine

③ Disregard the AI's result and re-examine

④ Stick to your original diagnosis

**C4.** If you use the AIADS in your diagnoses, do you trust its diagnostic results?

① Strongly trust (skip to C4-1) ② Trust (skip to C4-1) ③ Uncertain (skip to C4-2)

④ Distrust (skip to C4-3) ⑤ Strongly distrust (skip to C4-3)

**C4-1.** The reasons you trust it are: **(Select all that apply)**

① Improve diagnostic accuracy

② Improve diagnosis speed

③ Reduce workload

④ Many colleagues are using it

⑤ Have used it before and found it very useful

⑥ Other: ________

**C4-2.** The reasons you are uncertain are: **(Select all that apply)**

① Have not used it personally and am uncertain about its diagnostic accuracy

② Have doubts about the product and worry about false advertising

③ Not familiar with this product

④ Not yet widely used

⑤ Not yet officially approved

⑥ Other: ________

**C4-3.** The reasons you do not trust it are: **(Select all that apply)**

① Worry about its diagnostic accuracy and potential misleading information

② Worry about data leakage and security issues

③ Have doubts about the product and worry about false advertising

④ Fear of being replaced

⑤ Concerns that over-reliance might affect my diagnostic skills

⑥ Not familiar with this product

⑦ Not yet officially approved

⑧ Other: ________

**C5.** Have you concerned that the AIADS might give incorrect diagnosis? (If you choose ②, skip to C6): ① Yes ② No

**C5-1.** On a scale of 1-100, how concerned are you? (The higher the number, the greater your concern): ______

**C6.** If the AIADS makes an error in diagnosis, who do you believe should be held responsible? (Please rank):

① Hospital ② Pathologist ③ AI development company ④ Other: ______

**D.** **Behavioral intention/Practice**

**D1.** Please evaluate your willingness to use the AIADS by selecting the option that best matches your actual situation: strongly disagree, disagree, neutral, agree, or strongly agree

| Items | Strongly disagree | Disagree | Neutral | Agree | Strongly agree |
| --- | --- | --- | --- | --- | --- |
| ① I will proactively use the AIADS in my daily work. |  |  |  |  |  |
| ② I am willing to spend time learning how to use the AIADS. |  |  |  |  |  |
| ③ I will make diagnostic decisions based on the assistance of the AIADS. |  |  |  |  |  |
| ④ When encountering complex or uncertain cases, I will proactively seek help from the AIADS. |  |  |  |  |  |
| ⑤ I am willing to recommend the AIADS to my colleagues. |  |  |  |  |  |

**D2.** Are you willing to use the "AIADS " for diagnosis in clinical practice?

① Yes ② No

**D2-1.** Why are you willing/unwilling? _______________

**D3.** Have you ever used AIADS in the field of pathology before participating in this survey?

① Yes (answer D3-1 to D3-5) ② No (skip to D3-6)

**D3-1.** Have you ever made an incorrect diagnosis based on the assistance of the AIADS in certain situations?

① Yes ② Not yet ③ Uncertain the final diagnosis

**D3-2.** When using the AIADS for diagnosis, do you consciously verify the AI's diagnostic results?

① Always ② Usually ③ Sometimes ④ Rarely ⑤ Never

**D3-3.** When using the AIADS for diagnosis, what is your diagnostic approach?

**① Rely on AI diagnosis:** Trust the AI's diagnostic results and make a final diagnosis directly based on it.

**② AI-assisted diagnosis:** Review the AI's diagnostic results and marked lesion areas, then conduct a detailed examination and evaluation, incorporating clinical experience and other diagnostic information to make the final diagnosis.

**③ Independent diagnosis followed by comparison:** First, make an independent diagnosis without referring to the AI's results, then compare it with the AI's diagnosis to verify your own diagnosis.

④ Other diagnostic approaches: _______________

**D3-4.** What issues do you encounter while using the AIADS? **(Select all that apply)**

① AI interferes with my judgment

② Insufficient accuracy

③ Unaccustomed to new reading mode (e.g., accustomed to microscopy)

④ Low diagnostic efficiency

⑤ High learning curve of the software

⑥ Complicated user interface and inconvenient operation

⑦ Quality issues with digital pathology slides

⑧ Lack of mobile (smartphone) support

⑨ Lack of feedback mechanism

⑩ Other: _______________

**D3-5.** When using the AIADS, do you find it helpful for reading slides?

① Yes (answer questions D3-5-1 to D3-5-3)

② No (answer questions D3-5-4 to D3-5-6)

**D3-5-1.** In what ways do you think the AIADS is helpful to you? **(Select all that apply)**

① Providing diagnostic results (normal/abnormal)

② Indicating specific diagnostic classifications and corresponding risk

③ Annotate abnormal cells/tissues

④ Improve diagnostic speed

⑤ Consistent diagnosis, enhancing diagnostic confidence

⑥ Inconsistent diagnosis, reviewing and checking the result

⑦ Other: _______________

**D3-5-2.** How helpful do you find the AIADS for slide reading? (Please rate from 0 to 10, with higher scores indicating greater helpfulness)


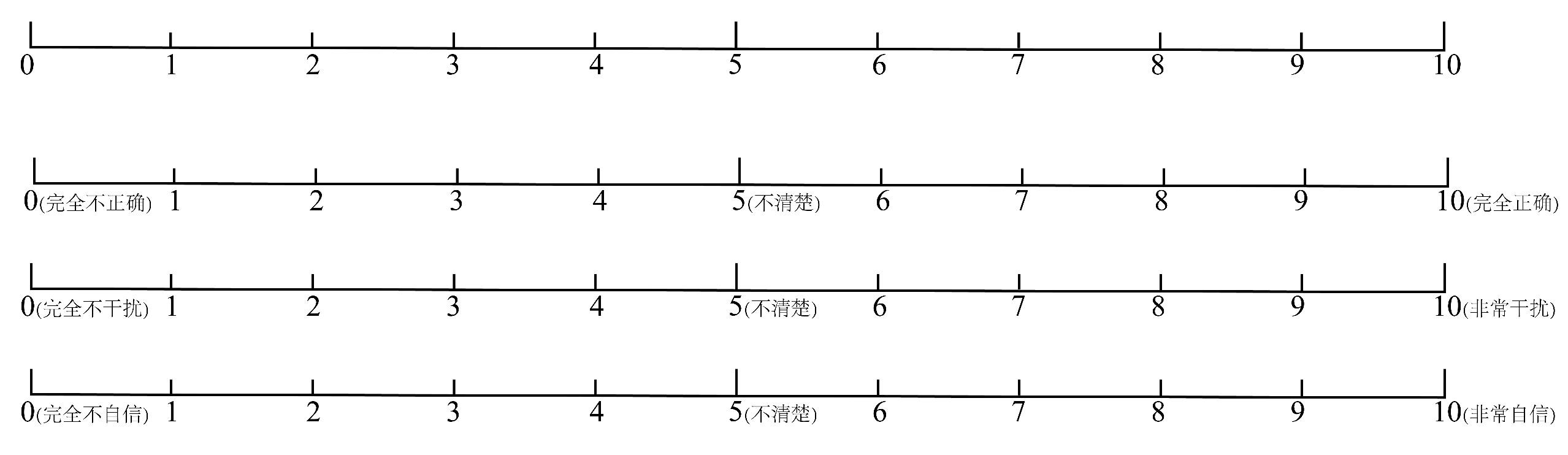


**D3-5-3.** How much has your diagnostic confidence improved with the assistance of AIADS? (Please rate from 0 to 10, with higher scores indicating greater improvement in confidence)


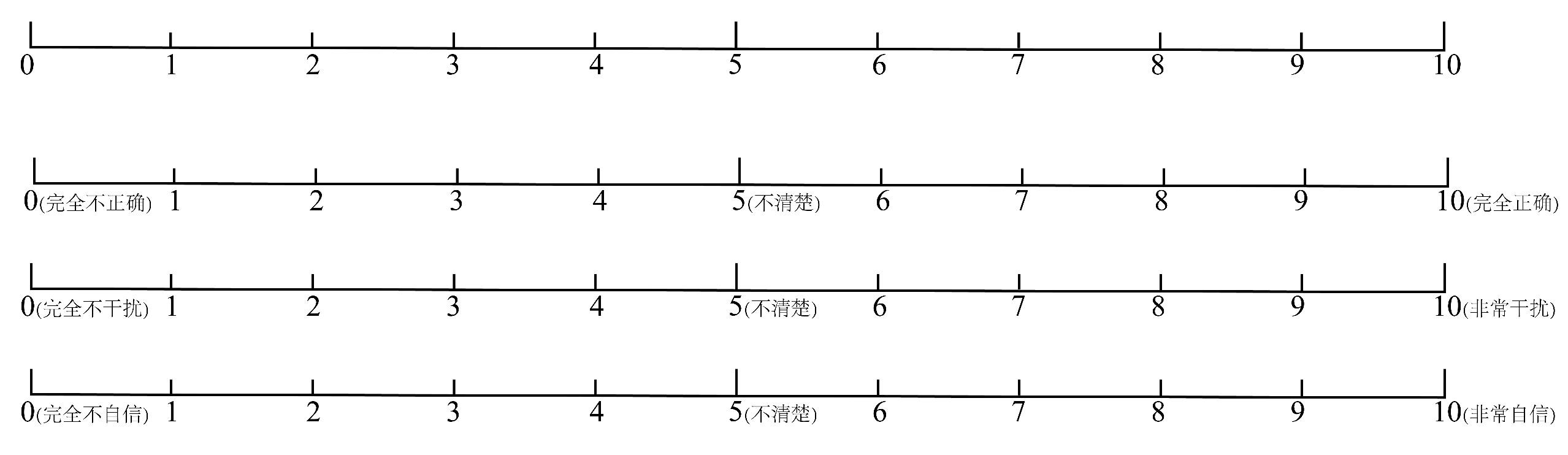


**D3-5-4.** Do you believe the AIADS's diagnoses are correct? (Please rate from 0 to 10, with higher scores indicating greater correctness)


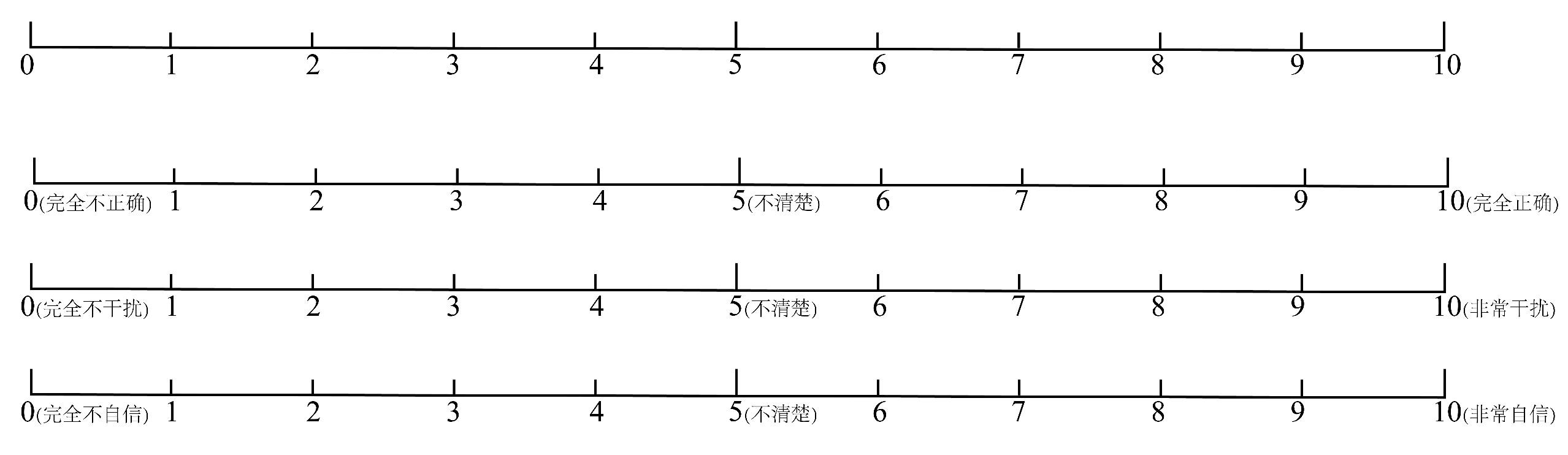


**D3-5-5.** Do you think the AIADS interferes with your diagnosis? (Please rate from 0 to 10, with higher scores indicating greater interference)


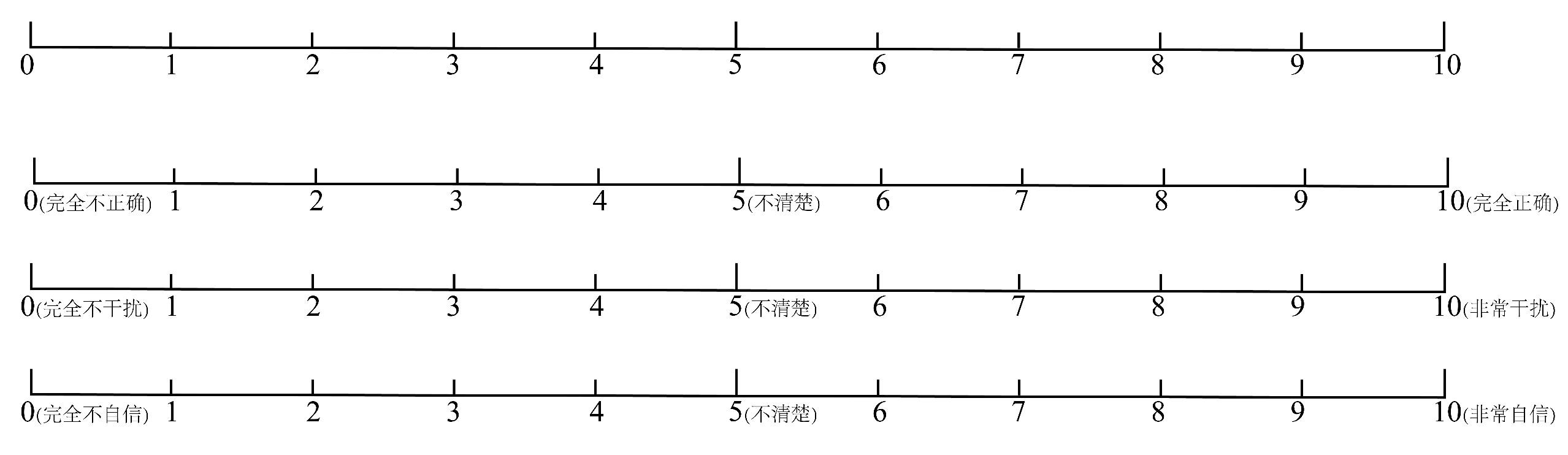


**D3-5-6.** How confident are you in your diagnostic results? (Please rate from 0 to 10, with higher scores indicating greater confidence)


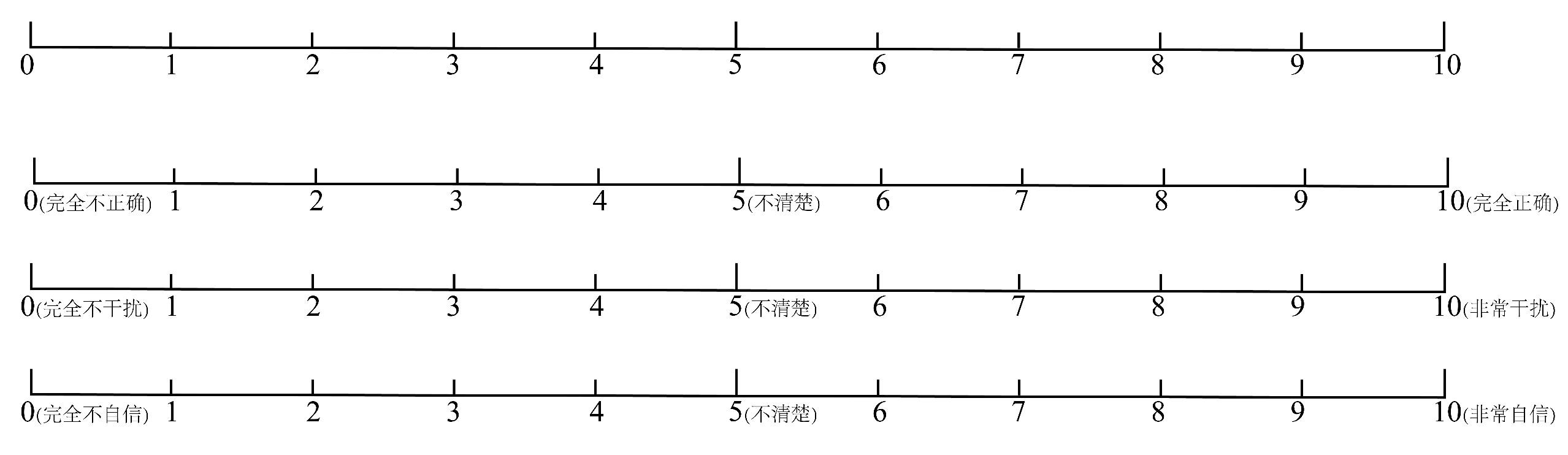


**D3-6.** When using the AIADS for diagnosis, what is your diagnostic approach?

**① Rely on AI diagnosis:** Trust the AI's diagnostic results and make a final diagnosis directly based on it.

**② AI-assisted diagnosis:** Review the AI's diagnostic results and marked lesion areas, then conduct a detailed examination and evaluation, incorporating clinical experience and other diagnostic information to make the final diagnosis.

**③ Independent diagnosis followed by comparison:** First, make an independent diagnosis without referring to the AI's results, then compare it with the AI's diagnosis to verify your own diagnosis.

④ Other diagnostic approaches: _______________

**D4.** What role do you think the AIADS should play in the diagnostic process?

① Primary decision-maker

② Assistive decision-maker

③ For reference only

④ Should not be used

⑤ Other: _______________

**D5.** What do you think are the main barriers to promoting the AIADS? **(Select all that apply)**

① Diagnostic performance do not meet clinical needs

② Courses/learning materials/professional guidance are not readily available, making it difficult to use

③ Lack of personal motivation/interest

④ Lack of support from hospital leaders/healthcare system leaders

⑤ Not yet officially approved

⑥ Other: _______________
