# Supplementary material for: A quantitative study of pathologists’ perceptions towards artificial intelligence-assisted diagnostic system
Source: PLOS Digit Health. 2025 Oct 17;4(10):e0001052. doi: 10.1371/journal.pdig.0001052 (PMC12533903; doi:10.1371/journal.pdig.0001052)

## S1 Fig. Geographic distribution of survey participants (N=224)

Note: This figure was created based on the standard map (Approval No. GS(2016)2880) downloaded from the Ministry of Natural Resources of China’s Standard Map Service website (http://bzdt.ch.mnr.gov.cn/).


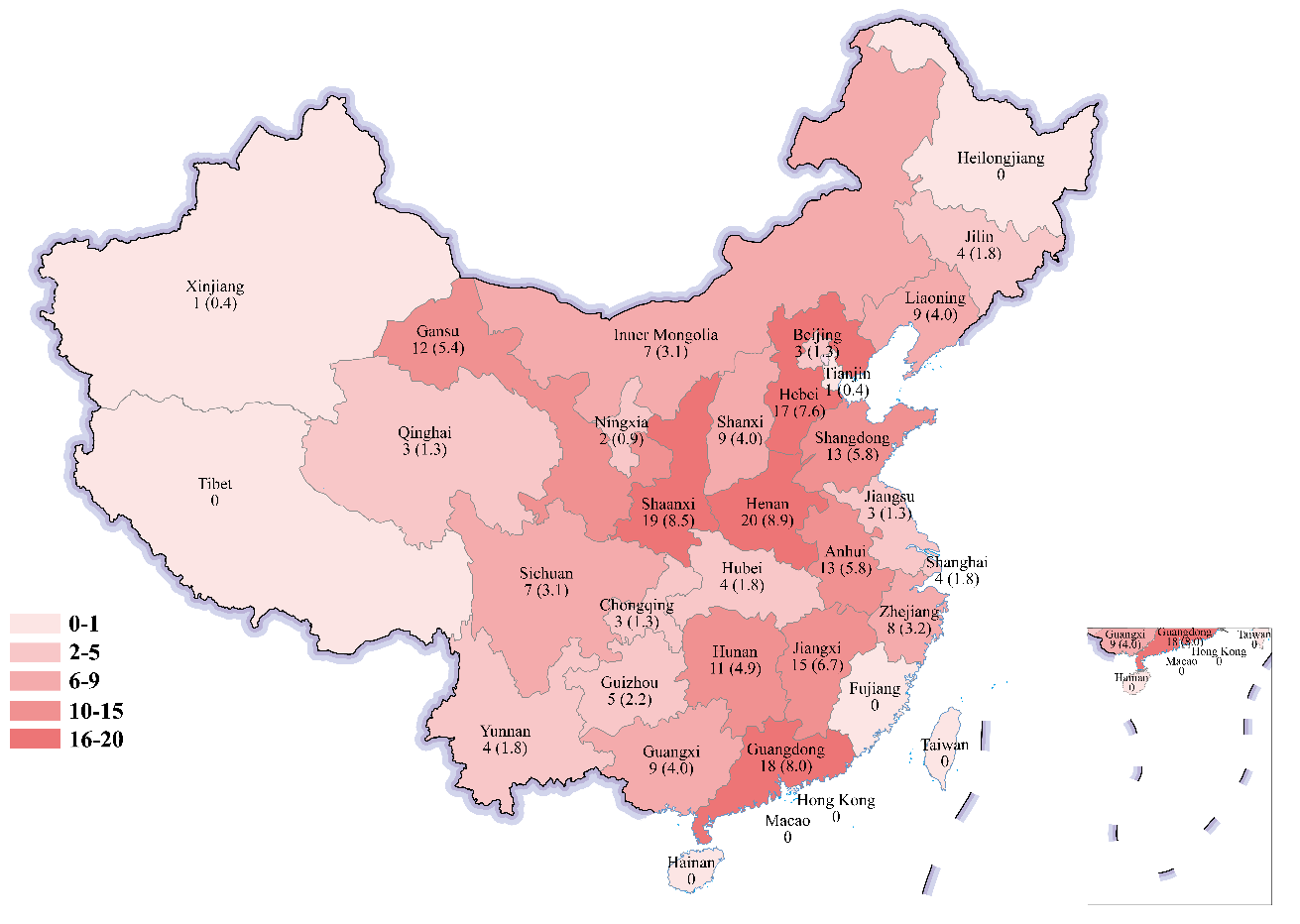

Supplement: S1 Fig — (DOCX) [file pdig.0001052.s002.docx]
