# Supplementary material for: A quantitative study of pathologists’ perceptions towards artificial intelligence-assisted diagnostic system
Source: PLOS Digit Health. 2025 Oct 17;4(10):e0001052. doi: 10.1371/journal.pdig.0001052 (PMC12533903; doi:10.1371/journal.pdig.0001052)
Supplement: S1 Table — (DOCX) [file pdig.0001052.s003.docx]

| **S1 Table.** The score of Likert scale about AIADS (N=224) | | | | | | | |
| --- | --- | --- | --- | --- | --- | --- | --- |
|  | **Items** | **Score** | **Very familiar** | **Familiar** | **Neutral** | **Unfamiliar** | **Very unfamiliar** |
| **Knowledge (3.42±0.97)** | |  |  |  |  |  |  |
| 1 | The function of AIADS. | 3.79±1.04 | 48 (21.4) | 126 (56.3) | 14 (6.3) | 27 (12.1) | 9 (4) |
| 2 | The working principle of AIADS. | 3.26±1.15 | 24 (10.7) | 99 (44.2) | 24 (10.7) | 65 (29) | 12 (5.4) |
| 3 | The usage methods of AIADS. | 3.44±1.13 | 31 (13.8) | 110 (49.1) | 21 (9.4) | 51 (22.8) | 11 (4.9) |
| 4 | The diagnostic performance of AIADS. | 3.38±1.10 | 25 (11.2) | 109 (48.7) | 27 (12.1) | 53 (23.7) | 10 (4.5) |
| 5 | The advantages of AIADS. | 3.67±1.05 | 37 (16.5) | 128 (57.1) | 16 (7.1) | 34 (15.2) | 9 (4) |
| 6 | The limitations of AIADS. | 3.45±1.09 | 27 (12.1) | 114 (50.9) | 24 (10.7) | 50 (22.3) | 9 (4) |
| 7 | Legal and ethical issues related to medical AI devices | 2.94±1.21 | 22 (9.8) | 69 (30.8) | 30 (13.4) | 80 (35.7) | 23 (10.3) |
|  | **Items** | **Score** | **Strongly agree** | **Agree** | **Neutral** | **Disagree** | **Strongly disagree** |
| **Attitude (3.48±0.44)** | |  |  |  |  |  |  |
| 1 | Using AIADS enhances my confidence in the diagnosis. | 3.78±0.71 | 28 (12.5) | 124 (55.4) | 68 (30.4) | 2 (0.9) | 2 (0.9) |
| 2 | I consider AIADS results trustworthy. | 3.49±0.73 | 17 (7.6) | 87 (38.8) | 110 (49.1) | 8 (3.6) | 2 (0.9) |
| 3 | I believe that AIADS may improve the performance of pathologists. | 3.77±0.72 | 25 (11.2) | 135 (60.3) | 53 (23.7) | 10 (4.5) | 1 (0.4) |
| 4 | I believe that AIADS may improve my work efficiency. | 4.15±0.71 | 68 (30.4) | 127 (56.7) | 26 (11.6) | 1 (0.4) | 2 (0.9) |
| 5 | I believe that AIADS will completely replace the diagnosis by pathologists. | 2.56±0.99 | 10 (4.5) | 27 (12.1) | 67 (29.9) | 95 (42.4) | 25 (11.2) |
| 6 | I believe that AIADS may reduce pathologists' professional abilities.* | 3.1±0.94 | 10 (4.5) | 73 (32.6) | 79 (35.3) | 54 (24.1) | 8 (3.6) |
| 7 | I believe that AIADS can offer a higher diagnostic quality than pathologists. | 2.98±0.94 | 11 (4.9) | 51 (22.8) | 96 (42.9) | 55 (24.6) | 11 (4.9) |
| 8 | I would like to receive assistance from AIADS, especially in cases where my diagnosis is uncertain. | 3.98±0.71 | 43 (19.2) | 142 (63.4) | 32 (14.3) | 5 (2.2) | 2 (0.9) |
| 9 | I am willing to adopt AI-assisted reading modes as a new approach to slides analysis. | 3.4±0.99 | 29 (12.9) | 79 (35.3) | 75 (33.5) | 35 (15.6) | 6 (2.7) |
| 10 | I believe AIADS should not be used in the field of pathology.* | 3.53±1.05 | 39 (17.4) | 86 (38.4) | 64 (28.6) | 25 (11.2) | 10 (4.5) |
| 11 | In my daily life, I trust AI devices that are intended to increase my personal safety. | 3.71±0.82 | 31 (13.8) | 114 (50.9) | 65 (29) | 11 (4.9) | 3 (1.3) |
| **Behavioral intention (3.47±0.44)** | |  |  |  |  |  |  |
| 1 | I will proactively use the AIADS in my daily work. | 3.96±0.75 | 50 (22.3) | 121 (54) | 49 (21.9) | 2 (0.9) | 2 (0.9) |
| 2 | I am willing to spend time learning how to use the AIADS. | 4.06±0.72 | 53 (23.7) | 139 (62.1) | 27 (12.1) | 2 (0.9) | 3 (1.3) |
| 3 | I will make diagnostic decisions based on the assistance of the AIADS. | 3.91±0.78 | 46 (20.5) | 122 (54.5) | 49 (21.9) | 4 (1.8) | 3 (1.3) |
| 4 | When encountering complex or uncertain cases, I will proactively seek help from the AIADS. | 3.91±0.80 | 46 (20.5) | 124 (55.4) | 44 (19.6) | 7 (3.1) | 3 (1.3) |
| 5 | I am willing to recommend the AIADS to my colleagues. | 3.99±0.73 | 49 (21.9) | 131 (58.5) | 40 (17.9) | 1 (0.4) | 3 (1.3) |
