# Supplementary material for: A quantitative study of pathologists’ perceptions towards artificial intelligence-assisted diagnostic system
Source: PLOS Digit Health. 2025 Oct 17;4(10):e0001052. doi: 10.1371/journal.pdig.0001052 (PMC12533903; doi:10.1371/journal.pdig.0001052)
Supplement: S2 Table — (DOCX) [file pdig.0001052.s004.docx]

| **S2 Table.** Subgroup analysis results of the Likert scale score for AIADS (N=224) | | | | | |
| --- | --- | --- | --- | --- | --- |
|  | **Items** | **Have you ever used AIADS in the field of pathology before participating in this survey** | | **T** | **P** |
|  |  | Yes (N=85) | No (N=139) |  |  |
| **Knowledge** | | 3.91±0.86 | 3.12±0.92 | 6.515 | <0.001 |
| 1 | The function of AIADS. | 4.21±0.86 | 3.53±1.06 | 5.246 | <0.001 |
| 2 | The working principle of AIADS. | 3.73±1.11 | 2.97±1.08 | 5.062 | <0.001 |
| 3 | The usage methods of AIADS. | 3.98±0.95 | 3.12±1.11 | 6.167 | <0.001 |
| 4 | The diagnostic performance of AIADS. | 4.00±0.89 | 3.01±1.05 | 7.589 | <0.001 |
| 5 | The advantages of AIADS. | 4.06±0.88 | 3.43±1.08 | 4.753 | <0.001 |
| 6 | The limitations of AIADS. | 3.98±0.90 | 3.12±1.07 | 6.421 | <0.001 |
| 7 | Legal and ethical issues related to medical AI devices | 3.41±1.22 | 2.65±1.12 | 4.652 | <0.001 |
| **Attitude** | | 3.55±0.50 | 3.16±0.54 | 5.265 | <0.001 |
| 1 | Using AIADS enhances my confidence in the diagnosis. | 3.82±0.85 | 3.75±0.60 | 0.716 | 0.439 |
| 2 | I consider AIADS results trustworthy. | 3.56±0.84 | 3.44±0.65 | 1.185 | 0.238 |
| 3 | I believe that AIADS may improve the performance of pathologists. | 3.80±0.75 | 3.76±0.70 | 0.450 | 0.653 |
| 4 | I believe that AIADS may improve my work efficiency. | 4.22±0.84 | 4.11±0.61 | 1.107 | 0.270 |
| 5 | I believe that AIADS will completely replace the diagnosis by pathologists. | 2.59±1.09 | 2.55±0.93 | 0.291 | 0.771 |
| 6 | I believe that AIADS may reduce pathologists' professional abilities.* | 3.14±0.98 | 3.08±0.92 | 0.472 | 0.638 |
| 7 | I believe that AIADS can offer a higher diagnostic quality than pathologists. | 2.99±1.02 | 2.98±0.88 | 0.076 | 0.939 |
| 8 | I would like to receive assistance from AIADS, especially in cases where my diagnosis is uncertain. | 3.99±0.85 | 3.97±0.61 | 0.160 | 0.873 |
| 9 | I am willing to adopt AI-assisted reading modes as a new approach to slides analysis. | 3.47±1.02 | 3.36±0.97 | 0.814 | 0.416 |
| 10 | I believe AIADS should not be used in the field of pathology.* | 3.60±1.12 | 3.49±1.00 | 0.769 | 0.443 |
| 11 | In my daily life, I trust AI devices that are intended to increase my personal safety. | 3.82±0.86 | 3.64±0.78 | 4.639 | 0.103 |
| **Behavioral intention** | | 4.02±0.84 | 3.93±0.58 | 0.908 | 0.183 |
| 1 | I will proactively use the AIADS in my daily work. | 4.09±0.84 | 3.88±0.68 | 2.119 | 0.035 |
| 2 | I am willing to spend time learning how to use the AIADS. | 4.05±0.91 | 4.06±0.57 | 0.161 | 0.872 |
| 3 | I will make diagnostic decisions based on the assistance of the AIADS. | 3.94±0.93 | 3.89±0.68 | 0.423 | 0.673 |
| 4 | When encountering complex or uncertain cases, I will proactively seek help from the AIADS. | 3.98±0.90 | 3.86±0.73 | 1.026 | 0.306 |
| 5 | I am willing to recommend the AIADS to my colleagues. | 4.06±0.90 | 3.95±0.61 | 0.986 | 0.326 |

* Assigning reverse scores to negative items.
