# Supplementary material for: A quantitative study of pathologists’ perceptions towards artificial intelligence-assisted diagnostic system
Source: PLOS Digit Health. 2025 Oct 17;4(10):e0001052. doi: 10.1371/journal.pdig.0001052 (PMC12533903; doi:10.1371/journal.pdig.0001052)
Supplement: S3 Table — (DOCX) [file pdig.0001052.s005.docx]

| **S3 Table.** The responses of participants who have used AIADS to behavioral intention/practice | | |
| --- | --- | --- |
| **Question** | **Answer** | **N (%)** |
| When using the AIADS, do you find it helpful for reading slides (N=85) |  |  |
|  | Yes | 83 (97.6%) |
|  | No | 2 (2.4%) |
| In what ways do you think the AIADS is helpful to you (N=83) |  |  |
|  | Providing diagnostic results (normal/abnormal) | 62 (74.7%) |
|  | Indicating specific diagnostic classifications and corresponding risk | 57 (68.7%) |
|  | Annotate abnormal cells/tissues | 78 (94.0%) |
|  | Improve diagnostic speed | 69 (83.1%) |
|  | Consistent diagnosis, enhancing diagnostic confidence | 57 (68.7%) |
|  | Inconsistent diagnosis, reviewing and checking the result | 62 (74.7%) |
| How helpful do you find the AIADS for slide reading (N=83) |  |  |
|  | Mean±SD | 7.80±1.45 |
| How much has your diagnostic confidence improved with the assistance of AIADS (N=83) |  |  |
|  | Mean±SD | 7.79±1.37 |
