# Supplementary material for: A quantitative study of pathologists’ perceptions towards artificial intelligence-assisted diagnostic system
Source: PLOS Digit Health. 2025 Oct 17;4(10):e0001052. doi: 10.1371/journal.pdig.0001052 (PMC12533903; doi:10.1371/journal.pdig.0001052)
Supplement: S5 Table — (DOCX) [file pdig.0001052.s007.docx]

## **S5 Table.** The result of direct effect and mediation effect

|  |  | Effect size | *SE* | 95%*CI* | Effect proportion |
| --- | --- | --- | --- | --- | --- |
| Model 1 |  |  |  |  |  |
|  | Total effect | 0.2108 | 0.0310 | (0.1498-0.2718) |  |
|  | Direct effect | 0.1750 | 0.0384 | (0.0994-0.2503) | 83.0% |
|  | Mediation effect | 0.0358 | 0.0168 | (0.0070-0.0719) | 17.0% |
| Model 2 |  |  |  |  |  |
|  | Total effect | 0.2304 | 0.0352 | (0.1610-0.3998) |  |
|  | Direct effect | 0.1879 | 0.0478 | (0.0974-0.2842) | 81.5% |
|  | Mediation effect | 0.0425 | 0.0186 | (0.0106-0.0832) | 18.4% |
| Model 3 |  |  |  |  |  |
|  | Total effect | 0.2421 | 0.0362 | (0.1707-0.3135) |  |
|  | Direct effect | 0.2106 | 0.0446 | (0.1272-0.2995) | 87.0% |
|  | Mediation effect | 0.0315 | 0.0223 | (-0.0107-0.0783) | 13.0% |

Model 1: No covariates were adjusted;

Model 2: Adjusted for gender, age, ethnicity, hospital level, education level, title, years doing pathology and specialized fields

Model 3: Adjusted for gender, age, ethnicity, hospital level, education level, title, years doing pathology, specialized fields and “Have you ever used AIADS in the field of pathology before participating in this survey”
