# Supplementary material for: A quantitative study of pathologists’ perceptions towards artificial intelligence-assisted diagnostic system
Source: PLOS Digit Health. 2025 Oct 17;4(10):e0001052. doi: 10.1371/journal.pdig.0001052 (PMC12533903; doi:10.1371/journal.pdig.0001052)
Supplement: S6 Table — (DOCX) [file pdig.0001052.s008.docx]

## **S6 Table.** Testing for Mediation model with subgroups

|  |  |  |  | M |  |  | Y |  |  | Y |  |  |
| --- | --- | --- | --- | --- | --- | --- | --- | --- | --- | --- | --- | --- |
|  |  |  |  | *Β* | *SE* | *P* | *Β* | *SE* | *P* | *Β* | *SE* | *P* |
| Model 1 |  |  |  |  |  |  |  |  |  |  |  |  |
|  | Have you ever used AIADS before being investigated | | | | | |  |  |  |  |  |  |
|  |  | No |  |  |  |  |  |  |  |  |  |  |
|  |  |  | X | -0.118 | 0.079 | 0.137 | 0.158 | 0.036 | <0.001 | 0.148 | 0.037 | <0.001 |
|  |  |  | M |  |  |  | 0.083 | 0.039 | 0.035 |  |  |  |
|  |  | Yes |  |  |  |  |  |  |  |  |  |  |
|  |  |  | X | 0.432 | 0.090 | <0.001 | 0.166 | 0.048 | 0.001 | 0.395 | 0.063 | <0.001 |
|  |  |  | M |  |  |  | 0.531 | 0.052 | <0.001 |  |  |  |
| Model 2 |  |  |  |  |  |  |  |  |  |  |  |  |
|  | Have you ever used AIADS before being investigated | | | | | |  |  |  |  |  |  |
|  |  | No |  |  |  |  |  |  |  |  |  |  |
|  |  |  | X | -0.048 | 0.079 | 0.556 | 0.165 | 0.037 | <0.001 | 0.161 | 0.041 | <0.001 |
|  |  |  | M |  |  |  | 0.084 | 0.041 | 0.062 |  |  |  |
|  |  | Yes |  |  |  |  |  |  |  |  |  |  |
|  |  |  | X | 0.4519 | 0.095 | <0.001 | 0.162 | 0.051 | <0.001 | 0.399 | 0.074 | <0.001 |
|  |  |  | M |  |  |  | 0.566 | 0.054 | <0.001 |  |  |  |

Note: X, Knowledge; M, Attitude; Y, Behavioral intention.

Model 1: No covariates were adjusted;

Model 2: Adjusted for gender, age, ethnicity, hospital level, education level, title, years doing pathology and specialized fields
