# Supplementary material for: A quantitative study of pathologists’ perceptions towards artificial intelligence-assisted diagnostic system
Source: PLOS Digit Health. 2025 Oct 17;4(10):e0001052. doi: 10.1371/journal.pdig.0001052 (PMC12533903; doi:10.1371/journal.pdig.0001052)
Supplement: S7 Table — (DOCX) [file pdig.0001052.s009.docx]

## **S7 Table.** The result of direct effect and mediation effect with subgroups

|  |  |  |  | Effect size | SE | 95%CI | Effect proportion |
| --- | --- | --- | --- | --- | --- | --- | --- |
| Model 1 |  |  |  |  |  |  |  |
|  | Have you ever used AIADS in the field of pathology before participating in this survey | | | | | | |
|  |  | No |  |  |  |  |  |
|  |  |  | Total effect | 0.1484 | 0.0365 | (0.0762-0.2206) |  |
|  |  |  | Direct effect | 0.1583 | 0.0365 | (0.0837-0.2254) | 106.8% |
|  |  |  | Mediation effect | -0.0099 | 0.0093 | (-0.0305-0.0060) | -6.8% |
|  |  | Yes |  |  |  |  |  |
|  |  |  | Total effect | 0.3948 | 0.0634 | (0.2688-0.5208) |  |
|  |  |  | Direct effect | 0.1656 | 0.0807 | (0.0182-0.3261) | 41.9% |
|  |  |  | Mediation effect | 0.2292 | 0.0693 | (0.0998-0.3743) | 58.1% |
| Model 2 |  |  |  |  |  |  |  |
|  | Have you ever used AIADS in the field of pathology before participating in this survey | | | | | | |
|  |  | No |  |  |  |  |  |
|  |  |  | Total effect | 0.1611 | 0.0405 | (0.0808-0.2413) |  |
|  |  |  | Direct effect | 0.1651 | 0.0448 | (0.0805-0.2577) | 102.5% |
|  |  |  | Mediation effect | -0.0040 | 0.0097 | (-0.0228-0.0187) | -2.5% |
|  |  | Yes |  |  |  |  |  |
|  |  |  | Total effect | 0.3991 | 0.0735 | (0.2524-0.5458) |  |
|  |  |  | Direct effect | 0.1621 | 0.0499 | (0.0624-0.2617) | 40.6% |
|  |  |  | Mediation effect | 0.2370 | 0.0774 | (0.0701-0.3814) | 59.4% |

Model 1: No covariates were adjusted;

Model 2: Adjusted for gender, age, ethnicity, hospital level, education level, title, years doing pathology and specialized field.
