# Supplementary material for: A quantitative study of pathologists’ perceptions towards artificial intelligence-assisted diagnostic system
Source: PLOS Digit Health. 2025 Oct 17;4(10):e0001052. doi: 10.1371/journal.pdig.0001052 (PMC12533903; doi:10.1371/journal.pdig.0001052)
Supplement: S8 Table — (DOCX) [file pdig.0001052.s010.docx]

## **S8 Table.** The Association Between AIADS usage and behavioral intention

|  |  |  | **B** | **S.E.** | **Wald** | **P** | **OR (95% CI)** |
| --- | --- | --- | --- | --- | --- | --- | --- |
| Have you ever used AIADS in the field of pathology before participating in this survey (Yes for reference) | | | | | | | |
|  | No | Model 1 | -0.243 | 0.294 | 0.681 | 0.409 | 0.784 (0.441-1.397) |
|  | No | Model 2 | -0.276 | 0.300 | 0.848 | 0.357 | 0.759 (0.422-1.365) |
|  | No | Model 3 | -0.254 | 0.316 | 0.647 | 0.421 | 0.775 (0.417-1.441) |
|  | No | Model 4 | 0.39 | 0.367 | 1.131 | 0.287 | 1.477 (0.720-3.033) |
|  | No | Model 5 | 0.172 | 0.347 | 0.245 | 0.621 | 1.187 (0.601-2.345) |
|  | No | Model 6 | 0.901 | 0.417 | 4.670 | 0.031 | 2.462 (1.087-5.573) |
|  | No | Model 7 | 0.386 | 0.341 | 1.280 | 0.258 | 1.472 (0.754-2.874) |
|  | No | Model 8 | 0.203 | 0.328 | 0.385 | 0.535 | 1.225 (0.645-2.329) |
|  | No | Model 9 | 0.915 | 0.392 | 5.436 | 0.020 | 2.496 (1.157-5.383) |

Model 1: No covariates were adjusted;

Model 2: Adjusted for ethnicity

Model 3: Adjusted for ethnicity, hospital level, title and years doing pathology

Model 4: Adjusted for ethnicity, hospital level, title, years doing pathology and knowledge

Model 5: Adjusted for ethnicity, hospital level, title, years doing pathology and attitude

Model 6: Adjusted for ethnicity, hospital level, title, years doing pathology, knowledge and attitude

Model 7: Adjusted for knowledge

Model 8: Adjusted for attitude

Model 9: Adjusted for knowledge and attitude
